# Exploring the behavioural determinants of compliance in resilient high-caries-risk patients who improved caries severity

**DOI:** 10.1186/s40359-024-02275-7

**Published:** 2024-12-23

**Authors:** Min Ching Wang, Ching Yi Wu, Wei Han Chen, Chieh Yu Liu, Yi Ching Ho

**Affiliations:** 1 Department of Dentistry, Taipei Municipal Wanfang Hospital (Managed by Taipei Medical University), No. 111, Section 3, Xing-Long Road, Taipei, 11696 Taiwan; 2 Department of Dentistry, National Yang Ming Chiao Tung University, Taipei, Taiwan; 3 Department of Stomatology, Taipei Veterans General Hospital, Taipei, Taiwan; 4 Institute of Oral Biology, National Yang Ming Chiao Tung University, Taipei, Taiwan; 5 Department of Speech Language Pathology and Audiology, Biostatistical Consulting Lab, National Taipei University of Nursing and Health Sciences, Taipei, Taiwan; 6 Department of Teaching and Research, Taipei City Hospital, Taipei, Taiwan

**Keywords:** Resilience, Compliance, Caries risk assessment, Protective factors, Caries severity, Children

## Abstract

**Background:**

The caries severity in childhood may predict caries conditions in the future and even in adulthood in caries risk models. Nevertheless, the rate of recurrent caries after treatment of severe early childhood caries is high and correlated with behavioural factors, rather than clinical indicators. Compliance with the caries control programme has been demonstrated to prevent root caries development in head and neck cancer patients, suggesting that compliance with treatment protocols is a more important key to bringing about successful outcomes than treatment protocols themselves. However, only few studies defined the triggers of compliance in patients with long-term successful treatment outcomes, especially in children. Furthermore, driven forces of compliant behaviours from patients’ aspects have not been described in the dental literature before. Regarding the need to improve current caries control interventions for children, in this study, behavioural determinants that shaped compliance of resilient children were investigated with a qualitative study design, for its advantage in revealing what an individual really feels which incorporates their experience without restriction from previous literature. Resilience was defined as improvement in caries conditions between primary and mixed or permanent dentitions.

**Methods:**

Interviews were performed with the patient group, including eight resilient children (M/F = 5/3) and their ten caregivers (M/F = 2/8), and the dentist group, including ten paediatric dentists (M/F = 6/4; clinical experience mean = 26.9 years, minimum = 16 years). Thematic analysis was used to identify main themes.

**Results:**

Four themes were identified: (1) dental things/teeth are their priority, (2) normalising, (3) tiger parenting/conscientiousness, and (4) trust. These determinants were identically described by both the patient and dentist groups. Dentists' suggestions were the priority, providing the norms in daily life of resilient patients and their caregivers. These patients found no excuses for failing to take dentists' advice, not only because they trusted their dentists, but also because they and their caregivers were conscientious about putting dentists' orders into practice.

**Conclusions:**

It is implementing suggested oral health behaviours daily, but not merely agreeing with professional advice, that alters the fate of teeth in these resilient patients.

**Supplementary Information:**

The online version contains supplementary material available at 10.1186/s40359-024-02275-7.

## Background

Children who have severe early childhood caries (SECC) are prone to have new caries [1]. Although cries risk remains the same as children age [2], SECC is not only a serious childhood problem but may also increase caries severity even after they grow older [3]. Thus, initial caries risk in childhood is a predictor of future caries conditions [4]. The recent guide for caries management has been shifted to caries risk assessment models. There are various forms of risk-based caries management, but they share similar caries risk factors. Harmful bacteria, low levels of saliva, intraoral infectious conditions and caries experiences are the most-mentioned risks [5, 6]. However, as social and behavioural factors also contribute greatly to oral health, the influence of behavioural factors has been underestimated in previous models for children [7].

Behavioural factors influencing children's caries risk, such as parental beliefs and compliance, are proven to influence caries relapse after dental care under general anaesthesia (DGA) [8, 9]. While caries recurrence rate after DGA was 37—54% in 6 months [10, 11], the non-compliant behaviour, defined by non-follow-up or irregular follow-up, is the strongest predictive factor of recurrent caries and repeated DGA [10, 12, 13]. Meanwhile, the relapse rate is not relevant to caries severity before treatments [13], caries prevalence, the level of bacteria [14], or the surface at risk after DGA [10], although these clinical caries indicators and caries history have been demonstrated as the most critical risk factors. Thus, in addition to identify risk factors of a child with SECC, “to establish an effective plan to promote protective factors with the aid of behaviour modification and to tailor the periodicity of oral evaluations” are crucial for caries management [15].

Compliance is defined as “the extent to which a person’s behaviour (in terms of taking medications, following diets, or executing lifestyle changes) coincides with medical or health advice” [16]. It has been demonstrated as a crucial behavioural factor in achieving effective and successful treatment outcomes. In the paediatric dental literature, therapeutic compliance has been mostly defined as regular follow-up [13]. Behaviour changes, such as increases in brushing frequency and reduction of eating/drinking frequency, etc., as a result of following professional suggestions that target at an individuals’ original risk factors, are also regarded as compliant behaviours that lead to long-term successful clinical outcomes [15, 17, 18]. Regarding that children with lower caries rate usually adhere to recommendations for preventive measures better [19–22], compliance is an important behavioural factor for caries prevention. Thus, facilitating children’s adherence to the regular dental preventive practices should be a part of the caries management protocol. However, in risk-based caries management, compliance has been underestimated and unclearly described in paediatric dental literature.

Behaviour changes were shown to be achieved more easily by interventions designed with behavioural determinants than non-theory-based interventions [23]. Regarding the role of compliance in caries prevention, to develop theory-based interventions that promote patients’ adherence to treatment plans, it is important to identify triggers of compliance in SECC children. Recent paediatric dental studies investigated factors that affect compliant oral health behaviours with the health belief model [24], the social cognitive theory [25] or the expanded theory of the planned behaviour model with the concept of sense of coherence (SOC) [17]. It was figured out that, in addition to certain socioeconomic factors, determinants for compliance included perceived susceptibility, perceived barriers, emotional coping and self-efficacy [24, 25]. Intention predicted the adherent behaviours of brushing or flossing [25], but not regular dental attendance [17]. These studies were conducted by analysing responses of school children, caries conditions and risks of whom were unknown, or their parents to behaviour questionnaire, however, lacking the correlation of compliant behaviours with successful treatment outcomes. Furthermore, dentists face individuals with SECC almost daily. They eager to design appropriate interventions that effectively change oral health behaviours and reduce caries occurrence specifically for these high-caries-risk children. Thus, it is important to know the driven force in children who have high caries risks but avoid the inevitable fate of the disease with behaviour models. However, behavioural determinants of compliance among these individuals might not be well recognized in recent literatures, since in previous studies, motivations were investigated in population with mixed caries risks. Thus, the aim of this paper was to explore determinants that shape compliant behaviours in resilient children who downgrade their caries severity. Considering what motivates patients’ compliance was not addressed enough in dental literature, interviews were adopted to enable participants to generate new insights freely.

## Methods

### Recruitment of participants

High-caries-risk resilient patients, whose caries severities were improved, and their caregivers were invited to discuss compliance or adherence from the parent's point of view. All of them were treated by the same dentist, MCW (first author) in Taipei Veterans General Hospital, which is a medical centre in the Capital of Taiwan. The included patients need to show both these following compliant dental behaviours:

1. Caregivers and children, who never lost to follow-up, had been followed up regularly for at least six years. Regular follow-up, which was confirmed with their dental attendance records, is the most repeatedly used objective measurement in compliance among dental literatures [13]. Since the high caries risk is defined as any caries experience including even one white spot lesion in the last three years [15], we extended the period of follow-up to 6 years to confirm the improvement of caries conditions in these children with high caries risk.
2. Caregivers and children showed active or positive attitudes towards complying with dentists and treatment plans. The therapeutic compliance dentistry is also defined as adhering to dentists’ recommendations and implementing them in a long-term [15, 17, 18]. Since dentists’ recommendations need to be tailored with individuals’ original risk behaviours [15], we did not strictly limit other types of oral hygiene practices that needed to be changed.

Since compliance with treatment protocols is different from cooperative dental behaviours [26], children who were not cooperative with pulp or extraction treatments and who had dental anxiety according to his/her own or caregivers or the dentist's observation were not excluded. 'Non-resilient' or 'refractory' high caries risk patients, who refused to participate this study or lost follow-up, were not included in this paper.

On the other hand, experienced paediatric dentists who had practised for more than 16 years were also recruited to discuss compliance or adherence among their high-caries-risk resilient patients from the clinician's point of view. The cut-off point for work experience as 16-year tenure was set based on following considerations. First, transition of full permanent dentition takes about 12 years for any individual. Gaining experiences of treating patients with high caries risks and having chances of observing their lifespan also needed time.

Ethical approval was obtained from the institutional Ethical Board (2019–09-010C). Informed consent was obtained for all participants including children. Written informed consent was obtained from the participants' parent/legal guardian/next of kin to participate in the study. Data analyses and collection were concurrent. Recruitment ceased after no additional information was generated from interviews and data saturation was reached [27]. A systematic review has highlighted that 9–17 interviews can reach data saturation [28].

### Definition of resilient high-caries-risk patients

In this study, 'resilient' high-caries-risk patients were those whose caries conditions in their mixed/permanent dentitions were improved when compared to their primary dentitions. Unfortunately, changes in caries severity from a long-term perspective among high-caries-risk patients could not be revealed by ICCMS™ or CAMBRA, since a patient is classified as a high-caries-risk patient when any white spot lesion or any previous restoration in recent years was detected [1, 5, 6]. In addition, the early loss of primary teeth and premature eruption of permanent teeth may not indicate a caries reduction. The recurrence of caries is also not clearly defined [5, 6]. To identify high-risk patients with improved caries severity, the w and W criteria were designed by experienced paediatric dentists and an oral pathologist to separately describe the worse clinical conditions in the primary and mixed/permanent dentitions (Tables 1 and 2). The rationale for these criteria were adopted from previous publications suggesting several factors for further definition of worse caries conditions. In addition to dmfs/DMFS, patients' caries conditions were also worse if affected teeth were newly erupted [29] or both anterior and posterior proximal teeth were affected [29, 30]. Caries patterns, anatomical sites [31–33], and severity measure of early childhood caries [34–36] were also adopted. Based on w and W criteria, 'resilient' high-caries-risk patients were those whose 'W' criteria were improved in mixed/permanent dentitions as compared to their 'w' criteria in primary dentitions.

**Table 1:** Worse conditions of high-caries-risk patients in primary dentition: w criteria

Conditions	Clinical indicators
High caries risk (w)	multiple anterior caries, or dmfs ≥ 8, or 2 - 4 crowns especially anterior ones
High caries risk (ww)	multiple posterior caries, or dmfs 12 - 24, or 4 - 8 crowns especially posterior ones
High caries risk (www)	multiple anterior + posterior caries, or dmfs > 24, or crowns > 8
**Note:**	
• Trauma is excluded in the dmfs	
• Early extraction due to caries is counted in the dmfs	
• Enamel hypomineralization or hypoplasia is counted in the dmfs	
• Possibly level up if the tooth surface show activeness, loss of luster or roughness	
• Possibly level up if plaque or roughness is noted on restorations	
• Possibly level up if signs of decalcification or caries are shown soon after eruption	

*dmfs* decayed, missing, filling, surface

**Table 2:** Worse conditions of high-caries-risk patients in mixed or permanent dentition: W criteria

Conditions	Clinical indicators
High caries risk (W)	recurrent pit caries of 6s, or DMFS≥8, or enamel hypoplasia or MIH
High caries risk (WW)	proximal caries of 6 s, or anterior teeth decalcification, or DMFS 12—16, or fracture of enamel hypoplasia or MIH
High caries risk (WWW)	anterior proximal caries, or caries or enamel hypoplasia of premolar, or DMFS > 16, or any pulp involvement or crown of any permanent teeth
**Note:**	
• Exclude trauma, dens invaginatus, or dens evaginatus oriented restorations	
• Possibly level up if criteria are noted before 7 s eruption	
• Possibly level up if the tooth surface show activeness, loss of luster or roughness	
• Possibly level up if plaque or roughness is noted on restorations	
• Possibly level up if restorations with initial signs of secondary caries, such as discolouration, or prone to loss	

*DMFS* Decayed, missing, filling, surface, *MIH* Molar incisor hypomineralisation

### Tool development

A topic guide for this semi-structured qualitative interview was developed based on a literature review (Supplemental S1) [2, 8]. Open-ended questions were designed with the theoretical domain framework (TDF) to comprehensively cover potential compliant or resilient behaviours and moderated by a paediatric dentist with qualitative research training (MCW). Some close-ended questions were used, when the participants, especially children or adolescents, did not know or want to explain their feelings and experiences. These questions might also base on what happen in the patients’ past dental histories, so the patients might just confirm what happen to them in these years. The TDF is derived from 33 psychological theories which are the most used ones in psychology, public health, and sociology. Using the TDF can prevent the omission of potential behavoural factors especially when the factors may not be revealed in the dental literature before [37]. Furthermore, there are behaviour change theories and techniques organising with the TDF. This would help further intervention design to be more effective and efficient [38]. The order in the TDF was not strictly followed to allow the participants to respond flexibly. The topic guide was piloted with other children to ensure that the questions were clear and understandable for this age group.

### Data collection

Interviews, as a qualitative study design, were used to explore the compliant behaviours in resilient high-caries-risk patients. Since there is little understanding of compliant behaviours, a qualitative study design is more suitable to probe the details at this stage and gives the participants freedom to express themselves, especially on their life experiences, thoroughly. A qualitative study can better accommodate nonlinear causality, which is common in psychology and behavioural science. People's thoughts and behaviours continually feedback to influence themselves and each other. The variables interact with each other across time which makes them seldom predictable in quantitative studies, but they can be organised in qualitative studies [39]. Individual interviews were chosen to protect individual privacy, especially patients' records [40]. All individual interviews were facilitated by MCW, who was the dentist and know what happened to the patients all these years. This can also protect the privacy of patients’ medical records. All interviews were audio-recorded, anonymised, and transcribed verbatim. The length of the interviews lasted 30 to 90 min.

### Data analysis

All interview transcripts were analysed using thematic analysis [41]. Analyses were undertaken with Microsoft Word. The coding was performed by the first author (MW) and the third author (WC). They translated the coding from Mandarin to English and confirmed them together. The themes were derived from the data, and the synthesis of the thematic map was achieved by three authors (MW, CW, and WC) to reduce interpreter bias from the first author [40]. The selection of quotes was to reflect the patterns of the data (themes). The most succinct quotes representing the themes were presented in Table 4 [42]. The second author (CW) read the coding and themes in English and confirmed them in Mandarin with the first author to ensure the translation quality. Considering the possible bias introduced during analyses by the researchers themselves, member-checking was undertaken during each session to confirm the interpretative validity [40]. Data source triangulation was also adopted to increase the internal validity. Different types of participants, children, caregivers, and other dentists, were invited to bring different perspectives on the research question [43]. This qualitative study was meant to study specific behavioural characteristics in this certain compliant population who reverse their caries severity. Hence, external validity (generalizability) may not be expected [44]. The paper was written with the COREQ guideline (Supplemental S2) [45].

## Results

### Overview of the participants

Two groups of participants were invited to participate in this study. There were eight children (M/F = 5/3) and ten caregivers (M/F = 2/8) in the patient group. All these patients, demographic data of which are shown in Table 3, received comprehensive dental restorative treatments in multiple outpatient department (OPD) appointments, and one of the patients received another surgical removal of mesiodens under general anaesthesia. There were ten dentists (M/F = 6/4; clinical experience means = 26.9 years, minimum = 16 years) in the dentist group. Seven of the dentists received their degrees from other countries. Seven dentists worked in both medical centres and private practice. Two dentists practised only in medical centres, and one dentist practised only in private practice.

**Table 3:** Demographic data of all resilient children

Pseudonym	dmfs + DMFS (age)	Decayed teeth	w criteria	DMFS (age)	Decayed teeth	W criteria	Frankl scale (19)	F/U
Celine	14 (2.4 y.o.)	ABs	w	8 (10.8 y.o.)	X (fissure sealants)	-	-- ➔ + +	8.3 yrs
Cole	15 (1.7 y.o.)	ABs	w	8 (11.5 y.o.)	6 s (Not recurrent)	-	- ➔ +	9.8 yrs
Che	20 (3.1 y.o.)	ABs	w	8 (13.7 y.o.)	6 s (Not recurrent)	-	- ➔ + +	10.6yrs
Chas	51 + 8 (7.5 y.o.)	Cs DEs, 6 s	www	8 (15.4 y.o.)	6 s (Not recurrent)	-	+ ➔ +	7.9 yrs
Cindy	32 + 8 (8.5 y.o.)	DEs, 6 s	www	8 (14.9 y.o.)	6 s (Not recurrent)	-	+ + ➔ + +	6.4 yrs
Clare	30 (2.7 y.o.)	ABs, DEs	www	8 (11.4 y.o.)	6 s (Recurrent)	W	-- ➔ --	8.8 yrs
Conan	38 (3.5 y.o.)	ABs, Cs DEs	www	8 (12.1 y.o.)	6 s (Recurrent)	W	-- ➔ --	8.6 yrs
Casper	61 (4.0 y.o.)	ABs, Cs DEs	www	10 (15.0 y.o.)	6 s (Recurrent) 12 s (decalcification)	WW	-- ➔ -	10.1 yrs
							**Average F/U yrs**	8.9 yrs

*dmfs DMFS,* Decayed, missing, filling, surface, *y.o.* years old, *F/U* follow-up, *yrs*. years

### Overview of the results

The themes from the compliance of patients who improved their caries risk were mainly related to patient aspects rather than dentist aspects and were especially related to caregiver aspects according to both dentist and patient participants (The patients' factors in this paper may include factors from children and caregivers afterward). Most of our dentist participants stated that the resilience of caries conditions depends on patient factors because dentists treated all their patients in the same way, but only a small proportion of patients were able to reverse their caries risk or conditions. Most of the dentists could clearly describe similar ideas and characteristics among the resilient patients, which were similar to the features of the included patient participants.

*[Doctor #8] I feel tiger moms who prefer to take control are more prone to be successful, but this is because these parents would keep on bringing their children back. They are not necessarily tiger moms. They care about dental things more. … I feel the ratio between dentists and parents is 3 to 7. 30% rely on dentists, and 70% rely on parents. … It is the parents who must accomplish things. They may not be control freaks. They may be determined. … Dentists provide solutions, but not all parents adopt the solutions.*

*[Doctor #7] Moms knew the 'Achilles' tendon' of their children. Their children's Achilles tendons were attacked, and then the children became compliant. … We originally thought the dentist, parents, and the child are a triangle, but it can be a linear relationship… Don't put the responsibility of controlling children on dentists…. You (dentists) need to persuade the parents to be willing to control their children.*

*[Doctor #9] The resilient patients who improve have parents who accept things very quickly.… When dentists tell them what to do, they (parents) just accept, and their habits change with your suggestions. Hence, the habit will be maintained after treatments, which means that these patients need only the first-round treatment. The non-resilient group has parents who have difficulties influencing their children. Children have caries in mixed dentition, and these children are not resilient.*

In the analysis, four essential themes were identified from the similar descriptions provided by dentist and patient participants: dental things/teeth as their priority, normalising, tiger parenting/conscientiousness, and trust. The subthemes and illustrative quotes in these four main themes are outlined in Table 4.

**Table 4:** Themes derived from the codings

Potential discussion point (subthemes)	Quotes
Theme 1: Dental things/Teeth are their priority	
• Parents aware of the importance of teeth from their experience	*[Researcher] You think teeth are important. For what reason?* *[Clare's male caretaker] Because my teeth are not good* *[Researcher] Thus, not because of the public media. Now, everybody always propagates tooth whitening and aesthetics* *[Clare's female caretaker] Tooth whitening is not that important. She should not have cavities and be normal. I don't think she should have tooth whitening*
• Parents aware of the dental severity of children	*[Doctor #10] But, I feel parents cannot catch (the warning from dentists), unless he has suffered what I (dentist) have warned. For example, children who have suffered from a toothache in the middle of the night or children whose faces are swelling or who have experienced cellulitis. … Parents can shock them by telling them that this thing can influence permanent teeth, so this may influence their behaviours. … Parents cannot understand until they see or witness it*
• Parents are willing to manage their time to visit the dentist. Nevertheless, parents may not treat their own teeth this way	*[Cole' male caregiver] Is it just the way, it is? We should come back when we have an appointment. That's the case. We make a day off when we are not available for his dental appointment. I think that is for sure. You should come back when dentists arrange visits, shouldn't you?* *[Cole' female caregiver] But, you may miss your own dental appointment*
• Parents do not care about their children's uncooperative behaviour or crying in the dental clinic because they want to make their teeth better	*[Researcher] So, were you so frightened after you saw her cry (during a dental treatment), that you would not come back?* *[Clare's female caretaker] It is much better than all her teeth rotting* *[Clare's male caretaker] I care of only her teeth and eyes. Only these two things. Other things are irrelevant*
Theme 2: Normalising	
• Brushing is a daily habit	*[Casper's female caretaker] … After he brushes his teeth by himself, he reminds me. He doesn't forget to brush his teeth. He brushes his teeth almost every time. Unlike us, we forget to brush when we are too tired. He has the habit already*
• Dental visit is a regular habit or routine. They do not even think about it, and they just do it	*[Chas] It is maintained as it used to be. … After a while, it is like a habit* *[Researcher] But, you don't think about it? Or, do you think a lot?* *[Chas] I don't think further*
• Parents do not see dental caries as a normal condition	*[Cindy's female caretaker] Nope, I like this way (treatment at the early stage) because, take her elder sister as an example, she got cavities over her anterior incisors. … The dentist (in the local clinic) felt it was normal that children have cavities. There is no need to be nervous or paranoid about cavities if the child is not in pain or experiencing swelling. …. I did not like these ideas (from the previous dentist). So, I searched for a dentist like you who prevents cavities from very early. I do not want to treat caries after they become severe*
Theme 3: Conscientiousness/tiger parenting	
• Authoritative parents or tiger parents who are determined can make children follow orders	*[Dentist #8] I think it (resilience) is tiger moms who like to control things. It brings success easier. Those who bring their children for follow-up appointments persistently are not necessarily tiger moms. They should care about these (dental things). Tiger moms tend to succeed. They might be rigid. … While some parents are harsh, they merely tell their children to do things in a harsh way, … Those parents shout out at their children, and then they turn their heads to cook. They do not always succeed, do they? … But, some parents talk to their children seriously, and they keep on persuading their children. They have a higher success rate, don't they? … The parents who want to achieve things but who may not be control freaks. They could be just determined*
• Parents usually implement their work carefully	*[Dentist #5] (Resilient parents) can’t lose. From the clinical aspect, sometimes, some parents help their children brush their teeth diligently, but their children still have caries. Although their children's caries is not very severe, the parents still feel devastated. So, I said, these parents want to be the No. 1. They think they get some knowledge of this world (dentistry), and they work hard to implement the knowledge. But if the results are not as expected, then resilient parents cannot accept it. Thus, this kind of parents wants to know 'why' when they discuss with dentists. After dentists analyse the factors for their children's caries, these parents can implement (a treatment plan)*
• Parents remember their dental schedule and details of treatment plans	*[Dentist #5] I think this group tends to remember the appointment time or the given treatment plan easily. These parents care about details, and they remember details. There is another kind of parent who you (dentist) must remind several times and, if you ask them every time, they (refractory parents) remember nothing. I think these things occur less in this (resilient) group*
• Parents like to be praised	*[Dentist #5] Most successful parents usually do not care how the child feels. These parents' goal is that the dentist gives them 100 points (A* + +*)* *[Researcher] Some of my resilient children cried a lot in the dental chair, but their parents just kept coming back for follow-up* *[Dentist #5] Therefore, the first thing is that the severity of caries made the parents take the dental thing seriously. The second thing is that these parents wanted to get 100 points, but their children did not want to get 100 points*
• Parents do not like to fail or miss things and do not like doctors to say bad things about them	*[Cole's male caregiver] … (About their experience in previous dental clinics) After the examination, the dentist condemned us (parents). He said that these were the parents' fault. … I felt sorry for my child. I wanted to find solutions when I was blamed. … After I was condemned then, I felt sad for a long time. I felt that I ignored these things and then made his teeth like this. Many people said, 'It's OK. It's just caries'. However, I felt depressed, and how come we didn't notice this*
Theme 4: Trust	
• Caregivers and children trust dentists' expertise or authority	*[Che] Is it normal to believe dentists? Instead of believing in myself, I would rather believe in a dentist*
• Caregivers and children respect dentists	*[Dentist #9] Parents would respect dentists, trust dentists, and have no negative feelings when receiving dentists' advice*
• Dentists are recommended by others	*[Celine's female caretaker] My friend recommended me to your clinic. … Then, I met you. The right one, saviour*
• Caregivers recommend their dentists to others	*[Celine's female caretaker] Many people ask me (which clinic is better), like other classmates' moms. I would just recommend your clinic*
• Believe the information from the dentist rather from the websites	*[Che's female caretaker] I googled information before I visit a dentist. After that, I followed the dentist's advice*

### Main theme 1: dental things/teeth are their priority

All the participants in both the dentist and patient groups thought that resilient patients who reversed their caries risk considered teeth to be the first priority. Resilient caregivers valued or appreciated the importance of dental health and the severity of the dental conditions of their children. They always remembered to visit their dentist regularly and were willing to take a day off to take their children to a dental appointment. Caregivers might obtain this attitude from their own experience, but they perhaps did not take their own teeth as their priority.

*[Researcher] …Is it inconvenient to keep asking for days off for dental treatments?*

*[Casper's female caretaker] Nope, I feel (dental) health is more important than work. I must do what needs to be done.*

*[Cindy's female caregiver] In addition, because my teeth are not well, I think teeth are very important.*

These caregivers valued dental treatment rather than their children's dental experiences. Although their children cried or fought during treatment procedures, behaviour management techniques or mild physical restraints were acceptable for them. None of our patient participants received restorative treatments under general anaesthesia or sedation. They understood that successful treatment outcomes might not come in an easy way.

*[Conan's female caregiver] Crying and fighting severely on the dental chair are normal responses of children. When they mentioned general anaesthesia, I denied it and wanted to have it step by step.*

*[Researcher] So you didn't want to have general anaesthesia?*

*[Conan's female caregiver] No. I wanted him to get used to it.*

*[Researcher] Is it inconvenient or difficult to come so many times?*

*[Conan's female caregiver] It's OK. We must be cooperative.*

*[Doctor #8] The chance for retreats is higher for TCI (a kind of sedation) children. … If the patient disappears, it is because of the attitude of the parents. Usually, when they come back, you will notice that it (caries severity) is the same for several years. Therefore, this is because of the attitude of parents. The second thing is how seriously they think about using fluoride. … Patients of TCI, honestly, are just the same as the study of GA. The record of the GA study shows a higher recurrent caries rate. If you do not perform behaviour management, after the treatment of TCI patients, they are still afraid of visiting dentists. After TCI, you can't train them for a while. Parents of these (non-resilient) patients hope that their children can visit dentists happily. When they know that you finish TCI, they will not let you touch their kids. In your mind, you will have a long window period. You cannot do a complete treatment.*

Although caregivers with a higher socioeconomic status had more resources to take better care of their children's teeth, but it was the caregivers’ compliance that make their children reverse their caries conditions. They recognised the importance of their children's dental health, and they were compliant with dentists to make their children's teeth better.

*[Doctor #6] I don't think socioeconomic status is the absolute factor. How parents value it (dental health) is more important. Very rich parents do not necessarily value it. An average family (with parents as office workers) might value it. However, among parents with high socioeconomic status, the ratio of this kind of people seems to be higher.*

*[Celine's female caregiver] I am the kind of person who gets a good thing and then promotes it. My friends (non-resilient parents) did not keep (visiting the dentist). I don't know what they were doing. They live truly close in an expensive area in the Capital and 10 minutes away from the dental clinic. … and these two moms did not need to work.*

### Main theme 2: normalising

It was normal or routine for all these resilient patients and caregivers to practise oral health behaviours, such as brushing teeth or dental visits. They, especially children, might not engage in health behaviours for any specific reason or motivation, such as making themselves healthier. They might maintain oral health behaviours because they got used to keeping on doing it or doing it subconsciously. They might also feel weird not doing it. That oral hygiene behaviours became their habits might be the reason why their oral health was maintained, and the effect could be long-term.

*[Cindy's female caregiver] I just tell her that you take good care of your teeth. It is for her own good, not for me.*

*[Cindy] … Because I have got used to keeping on coming back, it is a part of my routines. I don't think anything special about the dental visit.*

*[Che's female caregiver] Do you brush your teeth more seriously before you go to bed, Che?*

*[Che] I don't know. I would just brush my teeth.*

### Main theme 3: tiger parenting/conscientious

Many resilient caregivers were tiger parents. They were determined, not caring whether their children could receive treatment cooperatively or calmy. They decided that their children should go through the treatments and followed the dentists' suggestions. They remembered to come back regularly. They remembered the details of the dentists' suggestions and their children's daily dental behaviours. Hence, they can confirm these with their dentists in latter appointments to ensure they have achieved dentists' suggestions. Nevertheless, these caregivers and their children might not have a clear goal themselves. They just followed the dentists' orders.

*[Celine's female caregiver] Only a tiger mom can make a child of this age follow the rules. Otherwise, when you tell her thoroughly, she will not understand.*

*[Researcher] … Because you believe that she has the ability to achieve it, and you trust her that she can do it when you ask, so you do this. Or, you don't think about it. You just set the rules and she has to make it. …*

*[Celine's male caregiver] Because you dentists make the rules, I think that I should cooperate. … In addition, she likes you. She did not resist you at all. The first or second appointment did not count because you were strangers. She is OK afterwards.*

*[Dentist #5] (Resilient) parents always come back. They must know how to take care of their children. They always know what happens to their children. They even ask me whether they should change to electric toothbrushes. Parents use all kinds of means to change their children's behaviours and to make this (dental thing) work. These parents return every three months, sharing their experiences and feedback with the dentists. So, I know that they are serious.*

Most caregivers liked to achieve something or be praised by their dentists, feeling guilty or bad when they knew that they had not yet done enough. They would find ways to manage their children to follow the dentists' orders. Nevertheless, they might not want to be the best or flawless. They just did not like failing (Table 4).

### Main theme 4: trust

Most caregivers mentioned that they trusted or respected dentists. Thus, instead of following what was shared by their friends or websites, they would believe what was instructed by dentists. Some of the caregivers even stated that it was fate to meet the dentist.

*[Chas's female caregiver] Someone suggested you (the dentist). Serendipity. I quite believe in fate. If it felt right, I had confidence in the dentist. Then, the trust would be there.*

*[Celine's female caregiver] I am not that keen on (googling the information). However, I absolutely believe in the profession. Yes, I trust in the dentist's profession, and my kid likes the dentist. I can arrange and come to the dental appointment, (so) why not?*

These caregivers also had trust in their children, and they believed their children could succeed. These caregivers usually had good relationships with their children and did not force them to do things. Therefore, they can cooperate well with their children and then follow dentists' instructions together.

*[Doctor #9] Yes, resilient parents trust both their dentists and children. As long as we do this, everybody will get better together, and things will get better. … I did not feel these parents force their children. I felt the resilient children usually had a good family relationship. …The mom and child both felt these dental things were what should be done together. The mom got a new concept, and the child accepted and did what was asked. The resilient parents did not force their children. Then, they happily came every three months for fluoride varnish. I feel this is the characteristic of resilience. … Parents who need to force their children, their caries severity did not improve.*

## Discussion

No single clinical intervention has been strongly shown to control caries effectively. A combination of several caries preventive methods, which should be tailored with individual needs, is thus required. Patients’ compliance is perhaps what makes the treatment plans can be implemented, resulting in effective outcomes [46]. Compliant behaviours, such as regular follow-up, could be a protective factor to help people who have severe caries histories develop fewer new incipient caries [12, 13, 47]. However, what contributes to compliant behaviours of those who reverse their fate of teeth has not been explored enough in the caries literature. The beauty of this study was the analyses of behavioural determinants of compliance in patients whose caries conditions were improved in the long term. These resilient children with histories of severe early childhood caries did have fewer new incipient caries than expected ones, according to clinical experience and the literature on caries risk assessment [12, 13, 47, 48]. The characteristics of patients at low risk were another story and hence were not included in this study. These behavioural factors addressed in the following sections may come from children but primarily their caregivers. We would mention them as patients' factors afterward.

Priority given to dental care was what made these participants compliant to dentists and improve their severe caries conditions in the long term. A previous study reported that parents' attitudes towards oral health practices and a lack of knowledge might not influence recurrent caries conditions [8]. Patients usually know that teeth and oral health are important, but this does not imply that they implement what is suggested by dentists every day. Parents even doubted the possibility of 'keeping teeth cavity-free' after GA in a study [8]. This implies that knowledge of oral health alone was not enough to change oral health and behaviours. Furthermore, non-resilient (refractory, relapse) parents did not receive advice from dentists, and they had lower self-efficacy for controlling their child's oral health [9]. In contrast, our resilient participants perceived the concept that teeth are more important than other important things. As a result, even against or ignoring children's original willingness, resilient caregivers eliminated barriers to complying with the dentists' orders.

Normalising was also an essential factor that made these patients resilient. This is more than believing that no caries is normal in life [9]. However, according to our participants, these patients and their caregivers were not resistant to dentists' suggestions, even if the suggestions were new or not easy to implement at first. These caregivers and their children did not take dentists' suggestions as the knowledge in textbooks. In contrast, the resilient patients absorbed and diffused the dentists' suggestions as the norms in life. Compliant with dentists’ suggestions became daily routines. Therefore, they could follow the behaviour as a habit and kept it for a longer term than others, suggested by our participants were followed up for more than six years, which was much longer than in previous studies [10, 11, 13, 14]. Furthermore, it is difficult to probe these norms with traditional questionnaires, which may be mixed up with a pure dental knowledge. In contrast, we used the semi-structure interview to reveal the real belief or practise in the participants' lives, and interviews do not restrict participants' ideas with researcher-designed questionnaires [9]. Interventions designed based on participants' ideas may bring better behaviour changes in the real world [23]. Therefore, using norm-based interventions to change patients' risky behaviours in caries management may have been underestimated and is worth further exploration.

Normalising in our paper may be slightly different from subjective norm in the Theory of Planned Behaviour (TPB) [49]. The normalising in our participants is incorporating dentists’ suggestions into daily routines or habits, while subjective norm is individual’s perception about oral health practices influenced by the judgment of significant others or peers. Whether their dentists could be the significant others worth further investigation. One potential intervention to strengthen this relationship should involve sharing dentists’ own daily oral health practices, such as brushing more than two times per day, as demonstration of what they consider a natural and essential part of their lives. Framing these habits as the norms of dentists’ own lives, rather than emphasizing the negative consequences of neglecting oral hygiene, such as the risk of cellulitis, could foster a stronger sense of trust and relatability. Thus, patients may view these habits as achievable and meaningful and are encouraged to adopt them as part of their own routines. This shift in framing could ultimately help patients more readily accept and integrate these practices into their own lives, promoting better adherence to oral health routines. On the other hand in a study using TPB combing with sense of coherence (SOC) to predict adherence to preventive dental visits [17] showed that the adherence was more closely related with mothers’ SOC than TPB, which instead, was linked the intention that did not predict this behaviour directly [17]. The link between SOC and dental attendance was still present even after socioeconomic adjustments [50, 51]. That echoed with this research, in which not all resilient participants have better socioeconomic conditions than refractory patients. Thus, the ability to take advantage of the available resources may be more important than the amount of available resources, in the respects of caries management. The association between SOC and compliance or compliant behaviours as a result of normalising worth further studying. This may reshape clinical consultation strategies in the future. For example, dentists might explore with families whether they have support networks, such as relatives, who could help ensure children attend their appointments. By identifying practical solutions and leveraging community or family support, dentists could help alleviate logistical barriers, making it easier for families to adhere to treatment schedules. This approach could foster a more sustainable model to oral health management.

The tiger parenting identified in our study was different from the original, harsh stereotype of a tiger mom from Chua's book [52]. Instead of being authoritarian, tiger parents were conscientious and authoritative to a certain extent. In contrast, non-resilient caregivers were usually permissive [9]. Tiger parents tended to achieve their goals. These caregivers did not fail dentists, thus making their children follow or implement the dentists' guidelines. This behavioural characteristic perhaps the reason why most themes and quotes in this study came from caregivers but not children. Low conscientiousness may also be a risk factor for periodontal treatments [53]. Nevertheless, for periodontal treatments, a patient and a dentist have a direct relationship. In contrast, for paediatric treatments, a tiger parent is more like a moderator between the dentist and the child. Furthermore, differences in protocols of periodontal and paediatric treatments might also make conscientiousness play different roles. This is worth further studying the underlying mechanisms from social network and social support perspectives.

Trust might be the most obvious factor in patients' compliance. However, good relationships with dentists and regular follow-up did not guarantee reverses of caries severity in the longer term. Trusting non-resilient caregivers might have other priorities in their lives. The non-resilient (relapse) caregivers who were willing to comply might not be able to make their children follow them and their dentists, when compared to resilient (no relapse) caregivers. These caregivers may have difficulties maintaining brushing and oral health-related behaviours if their children resist caregivers' rules and structures. The communication of families with high-caries-rate children may not be well [54]. They may concern that forcing their children to dental clinics or brushing may harm their relationship with their children, which is more important than their children's teeth [9]. These non-resilient caregivers would rather see their children's teeth rotten, though they understand the consequences of caries and have personal trust relationships with dentists. Therefore, trust, friendliness, and good dental knowledge may be seen in the compliance of non-resilient patients. What makes our participants resilient are their other behavioural determinants discussed previously, i.e., priority, normalising, and conscientiousness, which should accompany with trust at the same time.

**Fig. 1:**
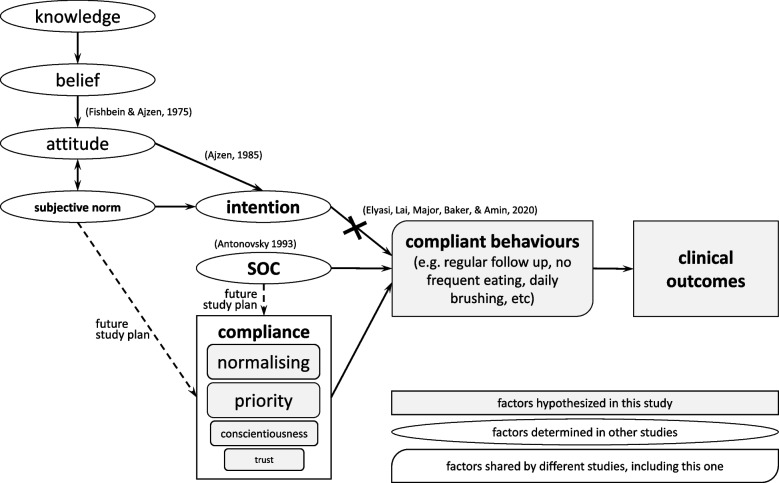
The thematic diagram to represent the analysis of the results: (1) dental things/teeth are their priority, (2) normalising, (3) tiger parenting/conscientiousness, and (4) trust. These four behavioural determinants shaped the compliance of resilient children who reversed their caries severity. The potential links between our findings and previous papers were also presented [16, 56–58]

The compliance in resilient patients from both our dentists' and patient participants' perspectives was very similar in all four themes, and this increased the internal validity of this study [43]. Resilience mainly resulted from the patients' own behavioural factors and characteristics. Our dentist participants expressed that the key was the patients and caregivers themselves rather than dentists. While the patient participants mentioned they were lucky to meet their dentists, however, they talked more about their own factors. Therefore, dentists may play a role as social support from the community [55]. This finding echoes previous papers, which reported that the compliance or attitude of patients, rather than treatment choices or caries severity, affected the treatment outcomes [10, 13, 14]. What change their teeth fate is definitely more than pure knowledge, which the refractory patients also obtained. The four behavioural determinants of compliance in our paper might not be directly linked with belief, attitude or subjective norm. Thus, relationship between the core components of Theory of reasoned action (TRA) [56], TPB [57], SOC [58] and the behavioural determinants of compliance worths further investigations. The theme trust may show their positive attitude or belief towards dental treatments. Resilient patients usually think complying with dentists is a good idea and will lead to fair dental outcomes. For example, the theme normalising may reflect the subjective norm influenced by dentists. The theme priority and conscientiousness may imply how an individual manages and implements a dentist’s suggestions, and shall be link with SOC [58]. Possible links between our findings and previous theories and potential future study plans was addressed in Fig. 1.

The clinical caries conditions and histories are the most important risk factors in caries risk assessment [2]. Hence, caries risk and severity need to be explored more at the same time. Even one caries lesion makes a patient as a high-caries-risk one [5, 6]. Among patients with high caries risks, improvement of their caries conditions could not be defined based on previous caries risk assessment tools, especially during mixed dentition. Additionally, our participants had much more severe caries conditions than previous papers. For example, in the paper of Isaksson et al. [3], the dmfs of their patients with manifest caries conditions was 3.5 at the age of three. The paper of Broadbent et al. depicted the caries trajectory pattern from five to 32 years old, and the mean DMFS of their ‘high’ trajectory group was about 4 at the age of nine [59]. In contrast, the dmfs and DMFS of our participants before treatment was 29.7 and 8, respectively. Hence, the w and W criteria were designed and applied here to help clinical dentists identify resilient patients, who do not fully recover but do improve their caries severity [29, 30, 32–36, 48]. The w and W criteria could not be validated due to our small size and need to be validated afterward with a larger group of data sources. Another limitation of this study is that we cannot compare the non-compliant patients who perhaps also reverse their caries severity because they are the patients who lost to follow-up. The dentist participants were hence invited to share their experiences with the non-compliant patients originally. However, all dentists do not have enough data about non-follow-up patients, and therefore, this paper could not address non-compliance. Future studies may explore the non-compliant patients with a population-level data source, such as National Health Insurance Research Database (NHIRD), to broaden our understanding of non-compliance.

The primary limitation of this study lies in the absence of refractory patients, a factor that may have introduced bias into our findings. Refractory patients—those whose caries conditions did not improve despite interventions—were not included in the study. These individuals can be broadly categorized into two subgroups: compliant and non-compliant, depending on whether they adhere to regular follow-ups or not. Understanding their experiences could have provided valuable insights, but challenges in recruitment meant their voices remain absent from this research. Compliant refractory patients often demonstrated trust in their dentists and a willingness to engage in care. However, they might face significant challenges in implementing recommended oral health practices. This difficulty may stem from a lack of conscientiousness or other personal or familial struggles. For instance, parents of these children might have found it challenging to enforce consistent oral hygiene routines, such as ensuring their child brushed twice daily. Some of these children might have some behavioural conditions such as impulsivity or attention deficit. Despite their willingness to cooperate in dental appointments, and some parents shared their struggles with dentists, they chose not to participate in interviews, possibly due to feelings of overwhelm or fear of judgment. This reluctance prevented us from exploring the deeper factors underlying their difficulties in overcoming caries, leaving a gap in understanding the barriers they face and their resilience—or lack thereof—in managing their oral health. Non-compliant refractory patients, on the other hand, presented a different set of challenges. Their irregular attendance at follow-ups often stems from socioeconomic or logistical hardships, such as an inability to afford treatment fees, difficulty taking time off work, or other pressing life circumstances. Many of these families likely wanted to prioritize their children’s oral health but found themselves constrained by circumstances beyond their control. Their irregular clinic visits made it exceedingly difficult to establish contact, let alone recruit them for participation for the study. This absence is particularly significant, as these individuals are often the most vulnerable and in need of targeted support. Without their input, we are left with an incomplete picture of how systemic issues like social inequalities intersect with oral health behaviours and outcomes.

Other limitations that might bias findings of this study was that the study was conducted by the treating dentist, whom the participants might felt that they could not decline. An independent researcher could increase the validity of the outcomes, of course. However, regarding that some discussions were private, an independent researcher might not be able to obtain trust between the familiar interviewer and responders. Additionally, the treating dentist is the one who know and understand what happened to these patients in these years. Familiar with these could help the interviewer to design some question based on participants’ past dental history, making adolescents speak, or sometimes it was difficult to keep the adolescents continue to speak about themselves with open-ended questions. Sometimes, their parents also would ask child-participants these leading questions because they had already known children’s answers. This may decrease generalizability of the study. We also ensured the patients that they can decline or withdraw the interview and this would not hinder their future treatment and the dentist-patient relationship. There was no resilient participant refuse to participate or dropout. Only one potential resilient participant who we discussed the initial idea of the study, but she lost follow up before the recruitment of the study. Hence, she did not meet the inclusion criteria which is regular follow-up. In addition, to increase validity of the study, two more researchers were involved in data analysis. Furthermore, anonymized responses were utilized to mitigate potential bias. Pseudonyms were assigned, and any details from the interviews that could reveal participants' identities or compromise their privacy—particularly sensitive medical records—were removed from the paper. These measures were implemented to protect participants and minimize bias.

## Conclusions

The behavioural aspects have been under-addressed in caries management literature. The prognosis of severe dental caries in children may not be totally determined by original caries severity, bacteria, and interventions. Thus, compliance with treatment protocols, which is likely a key to improving caries severity, was analysed in resilient participants defined by newly designed w and W criteria. Compliance does not merely rely on trust but, more likely, on the priorities of these patients and their caregivers. Implementing dental plans as their norms in life, perhaps with the spirit of tiger parenting or conscientiousness, also leads to better long-term outcomes. Thus, including a protocol to enhance compliance in caries management deserves an emphasis in clinical practice in the future.

## Supplementary Information

Supplementary Material 1.

Supplementary Material 2.

## Data Availability

The datasets analysed in the current study are available from the corresponding author on reasonable request.

## Abbreviations

DGA: Dental treatment under general anestheisa
dmfs: Decay-missing-filled surfaces (for primary teeth)
DMFS: Decay-Missing-Filled Surfaces (for Permanent Teeth)
DMFS: Decayed, Missing, Filling, Surface
GA: General anesthesia
SECC: Severe early childhood caries
TCI: Target-controlled infusion
TDF: Theoretical Domains Framework
NHIRD: National Health Insurance Research Database
