# Supplementary material for: Exploring the behavioural determinants of compliance in resilient high-caries-risk patients who improved caries severity
Source: BMC Psychol. 2024 Dec 23;12:774. doi: 10.1186/s40359-024-02275-7 (PMC11664850; doi:10.1186/s40359-024-02275-7)
Supplement: Supplementary file 1 — Supplementary Material 1. [file 40359_2024_2275_MOESM1_ESM.docx]

**Supplementary material S1**

**Topic guide**

1. In your opinion or idea, what are the causes and reasons for the improvement of the caries severity? The charteristics of behaviours?

2. What have you done to improve your caries severity? What are the interventions or treatments?

3. Why do you want to try so hard, and spend so much time improving your caries?

4. Is your child can accept or follow your or Dr's advice when he or she is so young? How do you regulate or manage their behaviour?

5. What are the facilitators and barriers to improving the caries severity?

6. How do you feel about the previous dental treatments and the improvement of the caries severity?

7. Where do you find the information about oral hygiene practices from dentists or bloggers?
