# Supplementary material for: Exploring the behavioural determinants of compliance in resilient high-caries-risk patients who improved caries severity
Source: BMC Psychol. 2024 Dec 23;12:774. doi: 10.1186/s40359-024-02275-7 (PMC11664850; doi:10.1186/s40359-024-02275-7)
Supplement: Supplementary file 2 — Supplementary Material 2. [file 40359_2024_2275_MOESM2_ESM.docx]

**Supplementary material S2**

**Supplemental table** Consolidated criteria for reporting qualitative studies (COREQ): 32-item checklist, based on Tong et al. 2007

| Domain 1: Research team and reflexivity |  |  |  |
| --- | --- | --- | --- |
| Personal Characteristics |  |  |  |
| 1 | Interviewer/facilitator | Which author/s conducted the interview or focus group? | The first author conducted all interviews. |
| 2 | Credentials | What were the researcher's credentials? E.g. PhD, MD | The author is a PhD student with 15 years clinical experience. Dental public health master degree. |
| 3 | Occupation | What was their occupation at the time of the study? | Clinical paediatric dentist and part-time PhD student. |
| 4 | Gender | Was the researcher male or female? | Female |
| 5 | Experience and training | What experience or training did the researcher have? | 15 years clinical work with high-caries-risk children. The thesis of the author’s master degree is a qualitative study, and the focus group was conducted under the supervision of the Psychological background teacher. Training of designing and conducting qualitative study in both Mandarin and English courses in Master and PhD programs. Published two qualitative studies and done three qualitative studeis |
| Relationship with participants |  |  |  |
| 6 | Relationship established | Was a relationship established prior to study commencement? | All the patients are the patients of the first author (MCW). This may reduce the differences of treatment outcomes from different dentists. These patients have known the researcher for more than 6 years.  All the dentists are the members of Taiwan paediatric dental association. The first author knew them in the association for several years. |
| 7 | Participant knowledge of the interviewer | What did the participants know about the researcher? e.g. personal goals, reasons for doing the research | All the participants knew the first author want to know why some patients can improve why most patients cannot. They knew the first author cannot get the answer from previous papers. |
| 8 | Interviewer characteristics | What characteristics were reported about the interviewer/facilitator? e.g. Bias, assumptions, reasons and interests in the research topic | The interviewer is the paediatric dentist of all patients. These patients may not able to tell their dentist all the truth, or they may be too similar. Therefore, other dentists were included to reduce the bias. |
| Domain 2: study design |  |  |  |
| Theoretical framework |  |  |  |
| 9 | Methodological orientation and Theory | What methodological orientation was stated to underpin the study? e.g. grounded theory, discourse analysis, ethnography, phenomenology, content analysis | Thematic analysis |
| Participant selection |  |  |  |
| 10 | Sampling | How were participants selected? e.g. purposive, convenience, consecutive, snowball | Purposive |
| 11 | Method of approach | How were participants approached? e.g. face-to-face, telephone, mail, email | face-to-face |
| 12 | Sample size | How many participants were in the study? | 28 participants |
| 13 | Non-participation | How many people refused to participate or dropped out? Reasons? | No |
| Setting |  |  |  |
| 14 | Setting of data collection | Where was the data collected? e.g. home, clinic, workplace | OPD clinic or Cafe. |
| 15 | Presence of non-participants | Was anyone else present besides the participants and researchers? | No, because there may be some patients’ privacy in the interviews. |
| 16 | Description of sample | What are the important characteristics of the sample? e.g. demographic data, date | The patients were all treated in medical centre rather private clinic.  Dentists who work in private clinics or medical centres were invited to bring the diversities from dentists’ perspectives. |
| Data collection |  |  |  |
| 17 | Interview guide | Were questions, prompts, guides provided by the authors? Was it pilot tested? | Topic guide was designed according to the theoretical domains framework and dental literature. It has been piloted with children of that age group. |
| 18 | Repeat interviews | Were repeat interviews carried out? If yes, how many? | No. |
| 19 | Audio/visual recording | Did the research use audio or visual recording to collect the data? | Audio recording |
| 20 | Field notes | Were field notes made during and/or after the interview or focus group? | Yes, field notes were taken during interviews. |
| 21 | Duration | What was the duration of the interviews or focus group? | All the interviews but one were longer than 30 mins. |
| 22 | Data saturation | Was data saturation discussed? | Saturation was reached. No new relevant knowledge was obtained between dentist and patient groups. |
| 23 | Transcripts returned | Were transcripts returned to participants for comment and/or correction? | No |
| Domain 3: analysis and findings |  |  |  |
| Data analysis |  |  |  |
| 24 | Number of data coders | How many data coders coded the data? | Two. The coding and the formation of themes were finished with the two researchers (MCW and WHC). |
| 25 | Description of the coding tree | Did authors provide a description of the coding tree? | Figure show the final themes. |
| 26 | Derivation of themes | Were themes identified in advance or derived from the data? | Derived from the data. |
| 27 | Software | What software, if applicable, was used to manage the data? | Word |
| 28 | Participant checking | Did participants provide feedback on the findings? | The latter participants feedback from the former participants in both dentist and patient group. |
| Reporting |  |  |  |
| 29 | Quotations presented | Were participant quotations presented to illustrate the themes / findings? Was each quotation identified? e.g. participant number | Yes. Demonstrated with their position. |
| 30 | Data and findings consistent | Was there consistency between the data presented and the findings? | Yes, the themes are similar in both patient and dentist group. |
| 31 | Clarity of major themes | Were major themes clearly presented in the findings? | Yes |
| 32 | Clarity of minor themes | Is there a description of diverse cases or discussion of minor themes? | Yes |
